# Microplastics and Nanoplastics in Cancer Progression: Biology and Public Health

**DOI:** 10.3390/biomedicines14010001

**Published:** 2025-12-19

**Authors:** Richard Ponce-Cusi, Claudia Barletta-Carrillo, Nesstor Pilco-Ferreto

**Affiliations:** 1 Escuela Profesional de Medicina, Facultad de Ciencias de la Salud, Universidad Nacional de Moquegua, Moquegua 18001, Peru; npilcof@unam.edu.pe; 2 Laboratorio de Genética Humana, Facultad de Ciencias Biológicas, Universidad Nacional Mayor de San Marcos, Lima 15001, Peru; cbarlettac@unmsm.edu.pe

**Keywords:** microplastics, nanoplastics, carcinogenesis, cancer progression, public health, public policies

## Abstract

Microplastics and nanoplastics (MPs/NPs), emerging as pervasive environmental contaminants, have raised growing concern due to their potential implications for human health. Among their diverse biological effects, recent evidence highlights their capacity to cross biological barriers, accumulate in tissues, and interact with cellular components in ways that may promote carcinogenesis. MPs/NPs can cause oxidative stress, inflammation, and epithelial barrier dysfunction, leading to cellular homeostasis disruption. Their interaction with endothelial cells and immune components further exacerbates pro-tumorigenic processes, including angiogenesis, immune evasion, and epithelial–mesenchymal transition (EMT), thereby potentially facilitating tumor initiation and progression. At the cellular level, these particles are internalized through various endocytic pathways, where they are associated with oxidative stress, inflammation, DNA damage, and barrier dysfunction—processes that have been linked to carcinogenesis. This review synthesizes current evidence on the cellular and molecular mechanisms through which MPs/NPs may contribute to cancer development, with particular emphasis on their interactions with endothelial cells and the tumor microenvironment. It highlights the need for further mechanistic and epidemiological studies to clarify the potential role of these particles in carcinogenesis. Given the increasing global production and environmental ubiquity of plastic particles, understanding their direct contribution to cancer development is critical for advancing both public health strategies and environmental regulations.

## 1. Introduction

Microplastics and nanoplastics (MPs/NPs), <5 mm in diameter and <1 mm in diameter, respectively, have become a ubiquitous and persistent environmental threat in the 21st century [1]. These particles originated from the fragmentation of larger plastic debris or are intentionally manufactured for industrial use [2,3]. Their small size and chemical stability enable widespread distribution across aquatic, terrestrial, and atmospheric ecosystems, where these particles resist degradation and accumulate over time, posing significant risks to ecological integrity and human health. The most prevalent polymers identified in environmental and biological samples include polyethylene (PE), polypropylene (PP), polyvinyl chloride (PVC), high-density polyethylene (HDPE), polyethylene terephthalate (PET), and polystyrene (PS) [4].

According to previous information, polyethylene (PE), one of the most widely produced plastics, is hydrophobic, chemically inert, and exhibits low density, making it highly buoyant and persistent in aquatic systems. Polypropylene (PP) shares similar hydrophobicity but has higher resistance to heat and chemical degradation, enhancing its environmental persistence [5]. High-density polyethylene (HDPE), a denser form of PE, is more rigid and durable, frequently used in packaging, containers and industrial insulation, and contributes significantly to plastic pollution due to its resistance to biodegradation [6]. Polyvinyl chloride (PVC), characterized by its chlorine content, has high density and mechanical strength; however, it can release toxic additives and plasticizers, raising concerns about its toxicity and interaction with living systems [3,6]. Polyethylene terephthalate (PET), commonly used in bottles and textiles, possesses high tensile strength and resistance to solvents, but its degradation releases micro- and nanofragments that can accumulate in tissues [2,4]. Finally, polystyrene (PS) is brittle and prone to fragmentation into MPs/NPs; its aromatic ring structure also facilitates sorption of hydrophobic contaminants, amplifying its toxicological relevance [7].

In aquatic environments, MPs/NPs contaminate several organisms such as plankton, fish, and shellfish, with escalating concentrations observed in coastal zones and open waters [8,9]. Sediment-dwelling organisms ingest these particles, facilitating their transfer through food webs. Terrestrial systems are similarly affected, with MPs/NPs entering soils via sewage sludge application as fertilizer and leaching into groundwater [10]. Atmospheric MPs/NPs, detected in urban and remote areas, further demonstrate their global dispersion [11].

Beyond their widespread distribution across aquatic, terrestrial, and atmospheric systems, the environmental persistence of MPs/NPs is attributable to their synthetic durability. As plastics fragment into smaller particles, their ecological impact intensifies rather than diminishes. This uninterrupted cycle of deterioration, spanning centuries, disseminates MPs/NPs throughout ecosystems, thereby intensifying their deleterious impacts. The documented impacts of these substances include physical harm to organisms ranging from plankton to mammals, chemical toxicity from leached additives, and ecosystem-wide disruptions [11]. Collectively, these factors highlight MPs/NPs as a multifaceted global crisis, with implications for biodiversity, ecosystem services, and public health.

Following their pervasive dissemination across multiple ecosystems, the toxicological behavior and environmental persistence of MPs/NPs depend on their intrinsic physicochemical characteristics. MPs/NPs are highly durable synthetic polymers with hydrophobic surfaces that allow them to adsorb a wide range of environmental contaminants [7,8]. Their small size enhances their mobility through environmental matrices and biological membranes, facilitating their entry into various organisms and tissues [9]. Particularly in the nanoscale, their high surface-to-volume ratio increases their chemical reactivity, cellular uptake, and potential for subcellular distribution, such as mitochondrial or nuclear localization [10,11].

Extending from these size-dependent interactions and surface properties, MPs/NPs are resistant to physicochemical and biological degradation, which contributes to their long-term environmental accumulation [12,13]. While this review focuses primarily on the plastic particles themselves, it is essential to acknowledge their dual role as both physical toxicants and vectors of adsorbed or embedded hazardous substances. These combined characteristics contribute to their capacity to induce oxidative stress, disrupt redox balance, interfere with cellular signaling, and promote chronic inflammation [14,15,16].

**Figure 1:**
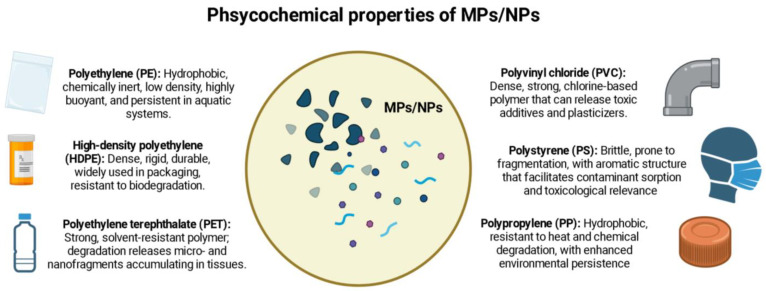
Physicochemical properties of MPs/NPs related to persistence, bioaccumulation, and toxicity. Created in BioRender. Ponce, R. (2025) https://BioRender.com/4szcpqa.

Due to their persistence, mobility, and bioactivity, MPs/NPs are emerging contaminants with complex ecological and health impacts. These features are summarized in Figure 1, which illustrates the molecular and environmental attributes associated with their toxicity and persistence.

## 2. Source of Exposure to MPs/NPs

### 2.1. Exposure Through Water and Food

The presence of MPs/NPs in water and food constitutes a significant pathway for human exposure, as evidenced by the documented detection in both drinking water and aquatic food chains [10]. Bottled water can contain hundreds of particles per liter. This exceeds the levels typically observed in tap water, potentially attributable to the degradation of packaging materials during storage and transportation processes [12]. It has been demonstrated that wastewater treatment facilities are not entirely effective in the removal of MPs/NPs, thereby enabling their continued presence in surface waters and potable supplies [9].

In addition to contaminated drinking sources, seafood constitutes a significant exposure vector, as marine organisms ingest MPs/NPs directly or through contaminated prey. Bivalves (e.g., mussels, oysters) pose heightened risks when consumed, including microplastic-laden digestive tissues [13]. These particles have been found to carry adsorbed toxicants, including persistent organic pollutants (POPs), which have been linked to an escalation in health risks [14].

Beyond marine-derived products, processed foods are increasingly recognized as contamination sources, with MPs/NPs having been detected in salt, sugar, and other staples due to production processes and packaging [15]. Furthermore, storage in plastic containers is a significant contributing factor, as PE and PP are known to degrade under the influence of heat or mechanical stress, resulting in the release of MP/NP fragments [16,17]. The ubiquity of MPs/NPs in essential resources underscores the urgency of improved filtration technologies and stricter regulations to mitigate exposure risks [14].

### 2.2. Airborne Exposure and Dermal Contact

Airborne MPs/NPs pose significant health risks through inhalation, with documented presence in urban, rural, and remote environments [10]. Urban areas are particularly affected due to high plastic consumption, vehicular emissions, and industrial activity, which contribute to airborne particulate matter [14]. These particles (<2.5 µm) can penetrate deep into alveolar regions, causing inflammation, oxidative stress, and respiratory dysfunction [18]. Chronic exposure is linked to asthma and COPD, while MPs/NPs (<100 nm) may cross the air–blood barrier, entering systemic circulation [19].

In addition to these pathways, dermal exposure occurs through contact with MP/NP-contaminated textiles, industrial materials, and personal care products [20]. Despite the skin’s capacity to serve as an effective barrier, the potential for penetration of the stratum corneum by MPs/NPs (<100 nm) remains a concern [21].

### 2.3. Bioaccumulation and Biopersistence in the Human Body

The presence of MPs/NPs in human tissues is a matter of concern, as it demonstrates bioaccumulation and biopersistence. This has significant implications for long-term health. Following entry via the respiratory or digestive systems, or through cutaneous absorption, these particles have been observed to evade conventional excretion pathways and accumulate in multiple organs, including the gastrointestinal tract, liver, kidneys, lungs, and bloodstream [22]. Their translocation across biological barriers occurs through passive diffusion and endocytosis, with MPs/NPs (<100 nm) being particularly capable of cellular internalization [23]. This occurs because synthetic polymers naturally resist enzymatic degradation. This is evidenced by the long-term persistence of common plastics such as PE, PVC, PP, HDPE, PET and PS in human tissues, despite prolonged exposure to hydrolytic and oxidative environments [24].

In parallel to their role as pollutant carriers, growing evidence indicates that the intrinsic physicochemical properties of MPs/NPs—such as size, shape, and surface charge—can directly affect cellular homeostasis and contribute to pathological processes [25]. These interactions can disrupt cellular balance through multiple mechanisms: altering signaling pathways, increasing oxidative stress, causing DNA damage, and impairing mitochondrial function. Particle characteristics critically influence these effects, with smaller MPs/NPs penetrating the blood–brain barrier and irregularly shaped particles demonstrating heightened surface reactivity due to increased surface area-to-volume ratios [26].

Increasing on these cellular-level effects, indicate a correlation between the accumulation of MPs/NPs and systemic health implications, including chronic inflammation, endocrine disruption, and immune dysfunction [27]. Addressing these risks requires multidisciplinary approaches combining advanced detection methods with mechanistic studies to elucidate particle–tissue interactions. Such research is essential for developing evidence-based strategies to mitigate exposure and associated health consequences [28] (Table 1).

## 3. Interest in the Relationship Between MPs/NPs and Cancer

The potential carcinogenic effects of MPs/NPs have garnered increasing scientific attention due to their pervasive environmental presence and ability to infiltrate human tissues. These particles demonstrate concerning bioaccumulation in critical organs including lungs, intestines, and circulatory systems, bypassing physiological barriers through multiple exposure routes [10,17]. These elements may promote carcinogenesis primarily through two mechanisms: the induction of chronic inflammation and genotoxic stress, processes that are closely associated with their physical characteristics and high surface interaction with biological structures [8,19].

In addition to these intrinsic carcinogenic mechanisms, MPs/NPs function as efficient vectors for toxic compounds, concentrating environmental pollutants on their surfaces and releasing them within biological systems [3]. This localized delivery of carcinogens promotes DNA damage while creating pro-tumor microenvironments through sustained oxidative stress and chronic inflammation [29]. Physical interactions further exacerbate these effects, as particle morphology influences cellular damage—irregular shapes and sharp edges mechanically injure tissues, perpetuating inflammatory cycles that precede malignant transformation [30].

Moreover, endocrine disruption represents an additional significant pathway, with MPs/NPs and their chemical additives interfering with hormonal regulation in sensitive tissues [19]. This dysregulation particularly impacts hormone-responsive cancers, altering proliferative signaling in breast and prostate tissues. Concurrently, MP/NP-induced reactive oxygen species (ROS) generation creates oxidative DNA lesions while impairing repair mechanisms, establishing conditions favorable for tumorigenesis [54,55].

**Figure 2:**
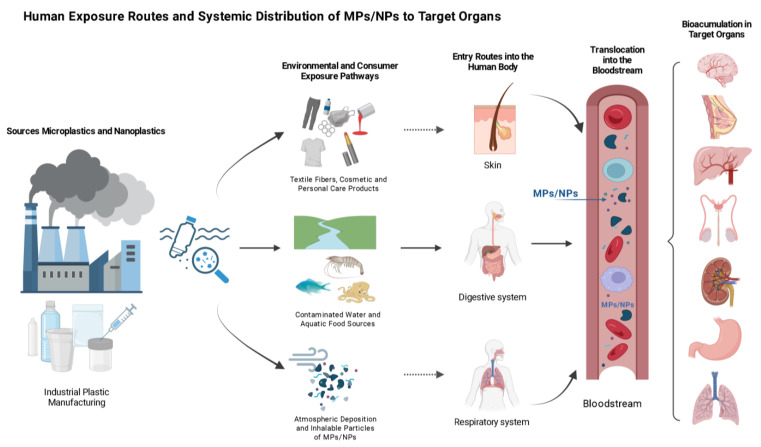
Schematic representation of the main exposure routes of MPs/NPs in humans. MPs/NPs enter the body through dermal contact (cosmetics, textiles), ingestion (contaminated food and water), and inhalation (airborne particles). These particles can translocate into the bloodstream and accumulate in critical target organs including the brain, adipose tissue, lungs, thyroid, kidneys, stomach, and intestines. This biodistribution raises significant concern for systemic toxicity and chronic disease risk, including cancer. Created in BioRender. Ponce, R. (2025) https://BioRender.com/4szcpqa.

Given these multifaceted oncogenic mechanisms, current evidence emphasizes the need for longitudinal studies to clarify dose–response relationships and tissue-specific effects [2]. As human exposure becomes increasingly unavoidable, understanding these carcinogenic pathways proves essential for developing targeted interventions and evidence-based policies to mitigate cancer risks associated with MP/NP pollution [17,54]. Figure 2 shows the sources of contamination, the route of entry into the human body, and the bioaccumulation in different organs.

## 4. Potential Mechanisms of Action in Carcinogenesis

### 4.1. Physical Effects of MPs/NPs on Tissues

The potential for MPs/NPs to induce cellular damage is multi-faceted, encompassing a range of biological effects including, lipid peroxidation, genetic alterations, MAPK pathway activation, membrane disruption, and mitochondrial dysfunction [56]. Tissue penetration is highly dependent on particle size: MPs/NPs (<100 nm) possess a high surface-to-volume ratio, enhancing their interaction with biomolecules and facilitating accumulation in organs such as the placenta [44]. In aquatic vertebrates, particles > 100 µm have been associated with abrasions, ulcers, and intestinal obstructions, while those <10 µm tend to persist in the lungs and promote chronic inflammation [57,58].

Within the range of particle-size-dependent effects, inhalation constitutes a primary route of exposure, with estimates suggesting that adults inhale approximately 53,700 MPs/NPs particles annually [58]. Particles < 10 µm can reach the alveoli, where their persistence alters epithelial integrity. In BEAS-2B cells, PS MPs/NPs (25–70 nm) decrease tight junction proteins (e.g., zonula occludens), increasing epithelial permeability and oxidative stress [59]. While fibers > 5 mm are typically cleared by mucociliary action, smaller nanoparticles evade this defense, accumulating in bronchioles and alveoli and contributing to fibrosis and pulmonary nodules [60,61].

Additionally, dermal exposure allows transcutaneous absorption of MPs/NPs (<100 nm) through hair follicles and sweat glands. Studies show selective internalization of 40 nm particles by Langerhans cells (CD1a+) [62]. Evidence from allergic dermatitis models further suggests that plastic-containing gloves and furniture materials can trigger chronic immune responses [63,64].

### 4.2. Chemical Cargo: MPs/NPs as Vehicles of Carcinogenic Pollutants

Despite their synthetic origin and chemical stability, MPs/NPs exhibit physicochemical characteristics—such as high surface area, hydrophobicity, and persistent polymeric structures—that promote their environmental accumulation and bioavailability. Their small size facilitates penetration into biological systems, where their surfaces can interact directly with cellular membranes, induce oxidative stress, and elicit localized inflammatory responses. Intrinsic properties, not external additives, are now seen as key factors in toxicity and cancer risk. Moreover, the surface characteristics of MPs/NPs may modulate their biological interactions by influencing protein adsorption and immune activation [65].

Due to these properties, ingestion is the main exposure route, allowing the direct interaction of MPs/NPs with gastrointestinal mucosa. This interaction facilitates particle retention and induces local biofilm formation, which has been associated with mucosal thinning and compromised intestinal barrier integrity [66,67]. Such alterations can enhance epithelial permeability and create a proinflammatory microenvironment conducive to neoplastic transformation. Mouse models of colon cancer reveal that exposure to MPs/NPs is associated with microbiota dysbiosis and distinct gene expression patterns in tumor-prone tissues, as evidenced by 16S rRNA and RNA-Seq analyses [68].

### 4.3. Inflammation

MPs/NPs induce inflammation primarily through ROS-mediated oxidative stress, which triggers cellular damage and the release of damage-associated molecular patterns (DAMPs). These DAMPs activate Toll-like receptors (TLRs), stimulating proinflammatory cytokine production (e.g., IL-6, TNF-α) and dysregulating innate and adaptive immune responses. Chronic exposure sustains this inflammatory state, impairing anti-tumor surveillance and promoting carcinogenic processes such as epithelial–mesenchymal transition (EMT) via STAT protein activation [69,70].

These mechanistic insights are supported by in vitro models using human intestinal cells (Caco2/HT29-MTX-E12) co-cultured with immune cells demonstrate that ingested MPs/NPs (50–500 μm) elevate proinflammatory cytokines (TNF-α, IL-8, IL-1β), albeit non-significantly [71]. Conversely, PVC exposure suppresses histamine release while upregulating IL-6 and TNF-α in other systems [72]. Murine studies have corroborated these effects, demonstrating that high-dose PE MPs/NPs alter gut microbiota diversity and amplify *TLR4/AP-1/IRF5* signaling, thereby driving intestinal inflammation [31].

Furthermore, MPs/NPs also exhibit systemic effects in C57BL/6J mice. PS particles have been observed to traverse the blood–brain barrier, resulting in a reduction in glial fibrillary acidic protein (GFAP) expression. GFAP is a marker of astrocyte activation, indicating neuroinflammation, particularly in younger mice [73]. Concurrently, hepatic immune markers show age-dependent alterations, suggesting tissue-specific susceptibility. In skin cancer models, MPs/NPs internalized by squamous cell carcinoma lines (SCL-1, A431) induce ROS-mediated mitochondrial dysfunction, opening permeability transition pores (mPTP) and activating the NLRP3 inflammasome. This cascade promotes inflammatory factor maturation, tumor proliferation, and genomic instability via oncogenic pathway dysregulation [32,74].

### 4.4. Oxidative Stress

MPs/NPs induce oxidative stress by disrupting cellular redox balance, primarily through excessive generation of ROS. Both intracellular and extracellular mechanisms contribute to ROS production, including mitochondrial electron transport chain activity, peroxisomal beta-oxidation, and oxidation of hemoproteins such as hemoglobin [75,76,77]. Antioxidant defenses, including superoxide dismutase, catalase, and glutathione-associated enzymes, work synergistically with Nrf2-mediated gene regulation to mitigate ROS. However, these systems are overwhelmed under sustained MP/NP exposure [78,79].

The toxicity of MPs/NPs is size- and concentration-dependent, with larger particles and higher doses exacerbating ROS generation. PS particles (5 µm, 50 g/L) disrupt mitochondrial membrane potential after lysosomal uptake, while plastic degradation byproducts further amplify oxidative damage via extracellular ROS pathways [80,81]. Mitochondrial dysfunction activates *MAPK* and *NF-κB* signaling, driving proinflammatory cytokine release and endoplasmic reticulum stress, as demonstrated in renal cells exposed to PS MPs/NPs [32,82]. Also, the MAPK pathway integrates oxidative stress responses, with ERK promoting survival and JNK/p38 triggering apoptosis. In murine models, MP/NP exposure increases p38 phosphorylation, correlating with oxidative stress levels and reproductive toxicity [38].

These findings underscore the dual role of ROS in initiating adaptive stress responses and perpetuating tissue damage, highlighting oxidative stress as a central mechanism linking MP/NP exposure to inflammation, organ dysfunction, and carcinogenesis [83].

### 4.5. Genotoxicity

MPs/NPs induce carcinogenesis through genotoxic and non-genotoxic pathways. Genotoxic pathways occur because of DNA damage via adsorbed mutagens (e.g., heavy metals, carcinogens) on MP/NP surfaces. Conversely, non-genotoxic mechanisms involve estrogenic compounds that bind cellular receptors, promoting proliferation and inflammation [65]. For instance, xenoestrogens from plasticizers may activate intestinal estrogen receptors, driving colorectal cancer progression.

Experimental support indicates genotoxic potential of MPs/NPs is well-documented. In human lymphocytes, PE particles (10–45 µm) increased micronuclei, nucleoplasmic bridges, and nuclear budding in cytokinesis-block micronucleus assays, indicating genomic instability without cytotoxic effects [84]. Similarly, PET-MPs/NPs (~136 nm) elevated ROS production by 30% and caused DNA strand breaks (0.10 lesions/10^6^ base pairs) in A549 lung cells via oxidative stress [45].

These observations highlight DNA damage mechanisms include direct physical interactions between MPs/NPs and genetic material, as well as ROS-mediated oxidative lesions (strand breaks, base modifications, crosslinks). These alterations disrupt tumor suppressor genes, DNA repair pathways, and apoptotic regulators, fostering carcinogenesis [9,85]. These findings underscore the dual role of MPs/NPs as physical and chemical genotoxic agents, emphasizing the need for further research into their interactions with cellular microenvironments.

### 4.6. Alteration of the Cellular Microenvironment

MPs/NPs ingested via contaminated food and water accumulate in the colon, disrupting the equilibrium between the intestinal microbiota and the mucosal barrier that protects colonocytes from luminal bacteria [65]. This disruption is exacerbated by the capacity of MPs/NPs to adsorb hydrophobic carcinogens, thereby increasing direct exposure of colonocytes to genotoxic agents. In murine models, PVC exposure induces gut dysbiosis, characterized by reduced beneficial taxa (e.g., Christensenellaceae, Akkermansiaceae) and enrichment of pathogens (Desulfovibrionaceae, Enterobacteriaceae), alongside MP/NP accumulation in tissues and weight loss. In addition, the co-culturing of artificial colon models with Caco-2 cells has provided further evidence that PE MPs/NPs compromise mucus integrity, thereby facilitating microbial translocation [86].

**Figure 3:**
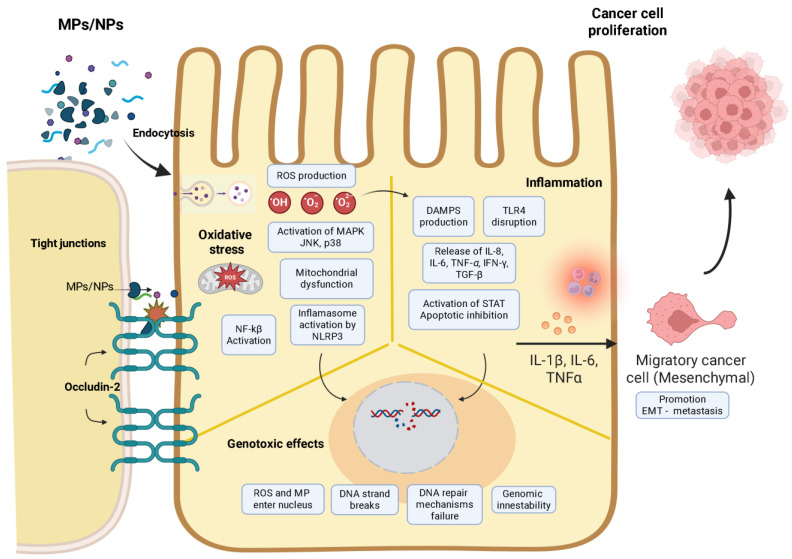
Schematic model illustrating cellular mechanisms of MP/NP-induced carcinogenesis. MPs penetrate the epithelial barrier and are internalized by host cells, where they initiate a cascade of harmful events. In the oxidative stress pathway, MPs/NPs trigger excessive production of ROS, leading to mitochondrial dysfunction, *NF-κB* activation, and inflammasome stimulation via *NLRP3*. In parallel, chronic inflammation arises from DAMP production and *TLR4* disruption, promoting the release of proinflammatory cytokines (IL-8, IL-6, TNF-α, IFN-γ, TGF-β) and activation of the STAT pathway, which inhibits apoptosis and supports tumor-promoting signaling. Simultaneously, genotoxic effects emerge as MPs/NPs and ROS reach the nucleus, inducing DNA strand breaks, impairing repair mechanisms, and fostering genomic instability. These converging processes facilitate EMT, cancer cell migration, and ultimately, tumor proliferation. Created in BioRender. Ponce, R. (2025) https://BioRender.com/4szcpqa.

Experimental in vitro models indicate that chronic PVC exposure in mice also impairs intestinal barrier function, driving metabolic dysregulation, lipid metabolism alterations, and systemic. The present findings underscore the dual role of MPs/NPs as both disruptors of microbial ecology and physicochemical mediators of mucosal damage. This dual role engenders microenvironments conducive to carcinogenesis through sustained inflammation and metabolic stress inflammation [87]. Figure 3 illustrates the molecular process associated with microplastic-mediated carcinogenesis.

### 4.7. Microbiome-Mediated Mechanisms

Emerging experimental and ex vivo studies indicate that ingestion of MPs/NPs consistently alters gut microbiota composition, typically reducing protective butyrate-producing taxa while enriching proinflammatory and genotoxic species such as pks^+^ *Escherichia coli* [65,88]. This dysbiosis compromises epithelial barrier integrity and activates innate immune receptors as TLR-4, leading to persistent low-level inflammation—a well-established tumor-promoting condition in colorectal tissues [89]. Moreover, microbial metabolic shifts observed in MP/NP-exposed models include increased colibactin and secondary bile acid production, both of which induce DNA damage and pro-proliferative signaling in colonocytes [46]. These microbiome-mediated pathways offer mechanistically plausible routes through which MPs/NPs may elevate colorectal cancer risk.

In addition, several pathways, such as oxidative stress and mitochondrial dysfunction, have been experimentally validated; others—including EMT modulation and microbiota-mediated genotoxicity—require further empirical substantiation. This highlights the need for future mechanistic and translational studies.

### 4.8. Disruption of Cellular Detoxification Mechanisms

Autophagy preserves intestinal homeostasis by maintaining mucosal integrity, modulating immunity, and regulating oxidative stress. Disruption of this process impairs the epithelial barrier and promotes chronic inflammation, contributing to inflammatory bowel disease [90,91]. MPs/NPs may interfere with autophagic pathways, exacerbating oxidative damage and inflammation and thus amplifying gastrointestinal vulnerability.

Numerous studies show that smaller MPs/NPs, especially PS, compromise epithelial integrity by disrupting tight junctions (TJs), key barriers that prevent harmful agents from entering systemic circulation [92]. Saha et al. (2023), using Caco-2 colonic epithelial cells, demonstrated that autophagy supports the proper localization of occludin—a key TJ protein—thereby reinforcing the epithelial barrier and mitigating inflammation [93]. However, autophagy induction by PS may also exert cytotoxic effects. Xu et al. (2023) showed that 100 nm PS triggers autophagy in both human intestinal cell lines and murine models, as evidenced by elevated LC3-II and p62 expression [94]. This reflects an impaired autophagic flux, resulting in lysosomal and autophagosomal accumulation.

Moreover, mitophagy, a selective form of autophagy targeting damaged mitochondria, is critical in esophageal tissue. Guanglin et al. (2024) reported that PS suppresses mitophagy in esophageal epithelial cells, leading to Fe^2+^ overload, elevated ROS production, and consequent cell death and inflammation in HET-1A and HEEC cell lines [95]. Autophagy also maintains respiratory epithelial homeostasis by supporting cell differentiation, mitochondrial quality, and inflammatory regulation, thus ensuring effective mucociliary clearance. During infection, it eliminates pathogens via xenophagy and restricts viral replication. Autophagic dysfunction in asthma and Chronic Obstructive Pulmonary Disease (COPD) leads to oxidative stress and epithelial damage. In this context, Annangi et al. (2023) demonstrated that intracellular PS in nasal epithelial cells increases ROS, impairs mitochondrial membrane potential, and markedly inhibits autophagic flux [96]. Yang et al. (2023) further observed that PS induces autophagy-dependent ferroptosis and ferritinophagy, impairing mitochondrial function and LC3-II levels [97].

MPs/NPs modulate autophagy via intricate signaling pathways that can be cytoprotective or cytotoxic, depending on physicochemical properties and cellular context. Initially, ROS generation by MPs/NPs activates the AMPK-mTOR-ULK1 axis, a key pathway for autophagy initiation, although lysosomal dysfunction frequently impairs flux completion [23,98,99]. Oxidative stress may also stabilize transcription factors that upregulate autophagy-related genes, yet without achieving full degradation. Importantly, MP/NP-induced inflammation appears tightly linked to autophagic modulation, with TGF-β1 and ferroptosis signaling pathways implicated. PS exposure increases intracellular iron and ROS, promoting cell death; impaired ferritinophagy further amplifies damage [97]. These findings underscore the dual role of autophagy in MP/NP exposure, governed by particle characteristics, exposure duration, cellular type, and autophagic competence [92].

### 4.9. Internalization, Toxicity, and Carcinogenesis in Endothelial Cells

Cellular internalization of MPs/NPs occurs mainly via endocytic pathways such as clathrin-mediated, caveolin-mediated endocytosis, macropinocytosis, and phagocytosis, with uptake determined by particle size and surface properties. MPs/NPs (<100 nm) may also cross membranes by passive diffusion. Small MPs/NPs (<0.1 µm) are internalized into lysosomes or the cytosol, whereas larger MPs/NPs (>1 µm) undergo phagocytosis or macropinocytosis [100,101,102,103]. Also, positively charged and functionalized particles show higher uptake and toxicity due to stronger interactions with negatively charged cell membranes [103,104].

These particles are internalized by endothelial and epithelial cells in a dose- and size-dependent manner, often via integrin α5β1-mediated uptake [40]. Once inside the cell, they accumulate and impair key functions such as angiogenic tube formation, wound healing, and migration, thereby compromising tissue integrity [105,106]. Internalization also triggers mitochondrial dysfunction, excessive ROS production, oxidative stress, and DNA damage—central events in carcinogenesis [105,106,107,108].

In addition, MPs/NPs disrupt tight junction proteins, increasing endothelial and epithelial permeability and promoting translocation across barriers [40]. This barrier dysfunction is accompanied by *NF-κB* activation, proinflammatory cytokine release, and ER stress, sustaining chronic inflammation [105,107,109,110]. Furthermore, MPs/NPs promote EMT through ROS and NOX4 signaling, enhancing cell migration, invasion, and metastatic potential [107,109]. The combined effects of cytotoxicity, inflammation, genotoxicity, and barrier impairment establish a microenvironment favorable for tumor initiation and progression [40,110].

## 5. Epidemiological Studies and Animal Models

### 5.1. Epidemiological Evidence

MP/NP contamination has emerged as a significant public health concern, with studies confirming their presence in human organs such as the lungs, blood, placenta, and intestines [111]. MPs/NPs have been detected in up to 90% of blood samples from healthy volunteers, with PE, PET, PS, and PP being the most frequently identified polymers [112,113]. A study of the olfactory bulb of deceased individuals in São Paulo, Brazil, revealed the presence of MP/NP particles in eight out of fifteen cases (53.3%), with the majority being PP (43.8%). These findings underscore the necessity for longitudinal studies to investigate the potential correlation between MP/NP exposure, neurodegenerative diseases, and environmental factors [114].

Variation in exposure levels is influenced by lifestyle and environmental factors, with higher concentrations of MPs/NPs reported in individuals who consume bottled water, seafood, or processed foods [115]. The presence of MPs/NPs in organs such as the prostate, liver, and kidneys—particularly in patients with chronic diseases like liver cirrhosis—suggests a potential tendency for accumulation in damaged tissues [116,117]. Although the long-term health implications remain uncertain, epidemiological data indicate associations between MP/NP exposure and systemic inflammation, metabolic dysregulation, and an increased risk of chronic diseases, including cancer [29].

Consistent with these patterns of accumulation and associated risks, humans are primarily exposed to MPs/NPs through ingestion of contaminated food and water and via inhalation, enabling the particles to penetrate biological barriers and accumulate in critical organs such as the lungs, liver, kidneys, and intestines, with some evidence suggesting their potential to reach the brain [35,114,118]. Certain MPs/NPs are capable of entering cells through endocytosis [117], and both in vitro and in vivo studies have demonstrated that these particles can induce inflammatory responses, cellular damage, and alterations in gene expression pathways involved in tumorigenesis. This evidence serves to reinforce the possible link between MPs/NPs and cancer development. Furthermore, MPs/NPs have been shown to persist within cells without being eliminated during mitosis, suggesting a potential for long-term cellular disruption [25].

Despite the accumulation of evidence, a pressing need persists for longitudinal epidemiological studies to elucidate the relationship between MP/NP exposure and cancer incidence in humans. This is an essential step for establishing causality and estimating relative risks [119]. The investigation of dose–response relationships is imperative for the establishment of safe exposure thresholds. Furthermore, certain occupational groups, particularly those in the textile and plastic industries, may be disproportionately vulnerable to microplastic-associated hepatotoxicity, particularly from exposure to PVC. This highlights the necessity of further research into the molecular mechanisms underlying potential carcinogenesis, including the role of genetic mutations and alterations within the tumor microenvironment [50].

### 5.2. Cancer Progression in Different Organs

MPs/NPs may contribute to cancer development. However, it should be noted that research in this area is still in its initial stages. Preliminary studies have begun to elucidate the biological mechanisms through which MPs/NPs may influence tumor progression, providing important but preliminary insights into their potential oncogenic roles [120].

#### 5.2.1. Prostate Cancer

Recent evidence has revealed the presence of MPs/NPs within human prostate tissue, highlighting a potential but still poorly understood link between environmental exposure and prostate health. Deng et al. (2024) conducted a comprehensive analysis of paired para-tumor and tumor prostate samples from 22 patients, identifying PET and PVC in both tissue types, with PS detected exclusively in tumor tissues, and reported a significantly higher MP/NP abundance in tumors [34]. Their findings also showed a predominance of irregular particle shapes, size distributions between 20 and 100 µm, and a positive correlation between tumor polystyrene levels and take-out food consumption. Complementarily, Demirelli et al. (2024) analyzed prostate tissue from 12 patients using microscopy and ATR-FTIR, detecting MPs/NPs in half of the cases, with particle sizes below 26 µm and diverse morphologies, the most common being polyamide (as nylon), alongside PP [116]. Both studies provide pioneering qualitative and quantitative evidence of MPs/NPs in the human prostate, underscoring the need for larger-scale investigations to clarify their role in prostate carcinogenesis.

#### 5.2.2. Breast Cancer

MPs/NPs have been implicated in promoting chronic inflammation and oxidative stress, processes known to facilitate tumor metastasis [37]. Studies have demonstrated that these particles can accumulate in solid human breast cancer [121]. Specifically, in vitro, PS MPs/NPs have been shown to be internalized by epithelial and breast cancer cells. Although no significant formation of colonies or increased cell fusion activity has been observed in breast cancer cell lines, mild stimulatory effects on cell proliferation and migration have been reported [122]. Moreover, in vitro, MP/NP exposure may inhibit the function of ATP-binding cassette (ABC) transporters and P-glycoprotein, both crucial elements of cellular detoxification mechanisms, potentially compromising cellular defenses and contributing to tumor progression [123].

In relation to these cellular uptake and detoxification effects, plasma protein-precoated polystyrene nanoparticles (PS-NPs) form a protein corona that modifies their cellular interactions, enhancing their uptake via endocytic vesicles and promoting intracellular localization within lysosomes. The modulation of protein corona formation may influence the biological effects of these nanoparticles on tumor cells, potentially mitigating disruptions in key signaling pathways involved in proliferation, including those related to the HER-2 receptor, which is critical for breast cancer cell survival and growth. Furthermore in vitro, PS has been shown to inhibit major signaling cascades, notably the phosphatidylinositol-3-kinase (PI3K)/protein kinase B (AKT) and mitogen-activated protein kinase (MAPK)/extracellular signal-regulated kinase (ERK) pathways, thereby directly impairing cell proliferation and viability [124]. In vitro research also suggests that MP/NP exposure may contribute to breast cancer progression through inflammatory responses triggered both in silico and in vivo, particularly involving interactions with estrogen receptor α (ERα) in MCF-7 cells. Additionally, plasticizing compounds derived from MP/NP production disrupt endocrine signaling pathways that regulate cancer cell proliferation, ultimately promoting tumor growth [125].

#### 5.2.3. Lung Cancer

The lungs are exposed to environmental microplastics (MPs) and may experience impacts from inhaling airborne particles. Recent findings from bronchoalveolar lavage (BAL) and blood samples of patients reveal novel insights into the potential link of microplastics with interstitial lung disease (ILD). Their presence in the respiratory system has been increasingly associated with pulmonary diseases, including ILD. Notably, MPs/NPs have been detected in BAL fluid from patients with ILD, especially those with fibrotic phenotypes, suggesting a possible role in disease progression [126]. An in vitro investigation on PS particles reveals that, at sizes of around 800 nm, MPs/NPs can penetrate human pulmonary epithelial cells (A549 cell line), inducing oxidative stress, cellular senescence, and apoptosis. These effects impair critical epithelial functions, augment basal inflammatory responses, and reduce tissue repair capacity after chronic exposure, thereby exacerbating long-term pulmonary conditions [23]. In vitro mechanistic studies further highlight that the internalization of PS is mediated by α5β1 integrin, facilitating nanoparticle entry and leading to mitochondrial dysfunction characterized by calcium-dependent depolarization and elevated ROS production. The subsequent oxidative stress, DNA damage, and necrosis compromise normal lung function and may contribute to the progression of fibrosis and other severe interstitial lung diseases [40].

#### 5.2.4. Cervical Cancer

The presence of MPs in the female reproductive tract has not been conclusively shown; however, hematogenous dissemination is considered a possible route of exposure in the human cervix. Studies in solid tumors have shown that certain MPs, particularly PE and PP, can enhance metabolic pathways involving amino sugars and nucleotide sugars, including D-mannose and cis-muconic acid, which may be implicated in cervical carcinogenesis [127]. Additionally, an association has been observed between MP/NP concentrations and patient age and body mass index (BMI), indicating that demographic and physiological factors may potentially influence MP/NP burden and, by extension, modify cancer risk [128].

#### 5.2.5. Gastric Cancer

The ingestion of MPs/NPs can alter the gastric microbiota, increase intestinal permeability, and release toxins that promote chronic inflammation and precancerous processes, even interfering with cancer therapy [129]. Experimental studies have demonstrated that PS MPs/NPs of 60 nm and 500 nm, when complexed with tetracycline, induce varying degrees of cytotoxicity in gastric cancer cells, characterized by reduced cell viability, elevated oxidative stress via ROS production, and apoptosis. Notably, 60 nm PS exhibits greater cytotoxicity than 500 nm particles, causing more pronounced DNA damage and upregulation of the pro-apoptotic marker Bcl-2-associated X protein (Bax) [130]. In colorectal cancer, plastics such as PVC, HDPE, and nylon have been identified as the most cytotoxic materials, with DNA damage in Caco-2 cells being particularly prominent after nylon exposure due to ROS generation [131]. Moreover, PS-MPs/NPs as small as 0.25 μm have been shown to enhance colorectal cancer cell proliferation in the human colorectal cancer cell line HT29, thereby increasing metastatic potential [25].

#### 5.2.6. Liver Cancer

The liver is a primary target organ for MP/NP accumulation, with exposure linked to hepatotoxicity and carcinogenesis. PS MPs/NPs in cell cultures and 3D models ranging from 1 to 10 μm can induce hepatotoxic and lipotoxic effects, reflected in cytotoxicity, disruption of lipid metabolism, ROS production, oxidative stress, and inflammatory responses. Increased expression of molecular markers such as hepatocyte nuclear factor 4 alpha (HNF4A) and cytochrome P450 2E1 (CYP2E1) further underscores the role of MPs/NPs in the development of hepatic steatosis, fibrosis, and potentially liver cancer [132,133]. Additionally, in vitro, the combination of PS with other MPs/NPs, such as triphenyl phosphate (TPHP) with a size of 0.07 μm, may exacerbate ROS-mediated damage, mitochondrial membrane depolarization, and lactate dehydrogenase (LDH) release, indicating a synergistic toxicity effect [42].

#### 5.2.7. Kidney Cancer

Recent research has demonstrated that the presence of PE and PS in the kidneys and urine can pose a risk to renal health, as MPs/NPs have the potential to circulate systemically and be filtered by the kidneys [134]. At lower concentrations, PS has been shown to stimulate proinflammatory cytokine release, while at higher concentrations, MPs/NPs induce autophagy and suppress inflammation through downregulation of NLRP3 inflammasome expression. However, these high concentrations compromise the integrity of the renal barrier by decreasing levels of critical proteins such as zonula occludens-2 and α1-antitrypsin, thereby increasing susceptibility to acute kidney injury [43]. Chronic accumulation of MPs/NPs in renal tissues promotes tubular cell damage, sustained inflammation, and impaired regenerative capacity, collectively elevating the risk of renal tumor development. Exposure to 1 μm PS in human kidney cell lines (HEK 293) induces morphological alterations, increased reactive oxygen species, and downregulation of key antioxidant and metabolic enzymes, suggesting that ingested MPs/NPs can cause cellular stress and metabolic disruption with potential adverse effects on human health [135]. Recent experimental evidence demonstrates that PS can directly impair human kidney development during early organogenesis; studies using kidney organoids show that exposure during the nephron progenitor cell stage induces oxidative stress, triggers apoptosis, and downregulates Notch signaling, ultimately leading to abnormal nephron structure and patterning [136].

#### 5.2.8. Nervous System

A preliminary investigation evaluated the effects of PE-MP (37–75 μm) on human glioblastoma (U87) cells using six concentrations (20 mg/mL to 0.62 mg/mL). The results demonstrated increased cell proliferation compared to untreated controls, indicating that MPs/NPs, as persistent environmental pollutants, might potentially aggravate cancer progression, particularly in aggressive malignancies like glioblastoma [36].

#### 5.2.9. Skin Cancer

Cutaneous squamous cell carcinoma (CSCC), a skin cancer with strong environmental risk factors, may be aggravated by MP/NP exposure. Studies in cell culture systems suggest that MPs/NPs enhance skin cancer cell proliferation through mitochondrial oxidative stress and NLRP3 inflammasome activation. Additionally, normal skin cells exhibit NLRP3-mediated inflammatory damage, implying that MPs/NPs could detrimentally affect both neoplastic and healthy tissues [32].

### 5.3. Detection and Characterization of MPs/NPs in Human Tissues

Although increasing experimental data supports the biological effects of MPs/NPs in cancer-related processes, validating their presence in human tissues remains a major analytical challenge. Various analytical techniques have been employed to identify and characterize these particles in biological matrices such as the placenta, lung, and gastrointestinal tissues, each with distinct advantages and limitations regarding resolution, particle size detection, and chemical specificity. For instance, micro-FTIR spectroscopy allows non-destructive polymer identification in tissues such as the placenta and lung, though it is limited to particles >10 μm [61,137]; micro-Raman spectroscopy offers high spatial resolution and detects particles <1 μm but is sensitive to fluorescence interference [112,138]; and pyrolysis–GC/MS provides high sensitivity and quantitative capabilities, albeit with the drawback of destroying the sample [139,140]. Additional approaches include SEM/TEM for nanometric imaging [141], flow cytometry for nanoparticle detection in liquids [142], PET imaging in animal models [143], and confocal laser scanning microscopy for 3D localization of labeled particles [32,144]. Table 2 summarizes the most relevant analytical approaches currently available for detecting MPs/NPs in human tissues, highlighting their applications and technical constraints.

In support of this need, recent in vivo and in vitro studies have begun to detect and characterize MPs/NPs directly within malignant tissues or cancer-related cell models. These findings provide emerging experimental evidence that links MPs/NPs to tumor biology across multiple organ systems. Table 3 summarizes documented cases of MPs/NPs identified in association with specific tumor types, including relevant polymer types, particle sizes, cellular targets, and mechanistic effects.

### 5.4. Research in Animal Models

Current research on the effects of MPs/NPs in cancer progression using animal models remains in its early stages; however, current findings have provided valuable insights into the mechanisms by which these materials may induce inflammation [151], oxidative stress [152], and tumor development. Most studies have centered on the gastrointestinal and respiratory systems, as they constitute the principal ingress and accumulation sites within the body. The MPs/NPs most commonly studied include PP, PS [153], PET [154], PVC [126,155], and polyurethane (PU) [126], selected for their environmental prevalence and widespread use in consumer products. Notably, investigations on PP have shown that particles ranging from 5 to 50 μm can be eliminated from the digestive tract within 24 h [156].

Focusing on gastrointestinal impacts, in murine models, dietary administration of PS MPs/NPs has been employed to investigate their effects on the digestive system. Chronic exposure has been shown to induce inflammation via TNF-α, IL-1β, and IFN-γ signaling. Concurrently, this exposure has been shown to cause dysbiosis of the intestinal microbiota, a condition that is linked to the disruption of intestinal epithelial barriers and an enhancement of intestinal permeability [157]. In vivo murine models have shown that exposure to 5–20 µm PS leads to excessive systemic accumulation, triggering severe inflammation and mechanical damage in organs such as the spleen, lungs, liver, and kidneys [158], whereas particles primarily led to significant intestinal barrier dysfunction and microbiota alterations [159].

In a transition from gastrointestinal to hepatic effects, in both in vivo and in vitro hepatic cell lines, PP MPs/NPs have been reported to disrupt lipid metabolism, a key factor in hepatocellular carcinoma development. Additionally, low doses of PP induce hepatotoxicity through oxidative stress, mitochondrial energy metabolism impairment, and dysfunction of genes related to nutrient homeostasis and NADH regulation. These effects, including the depletion of adenosine triphosphate (ATP) and the reduction in mitochondrial membrane potential, may compromise cellular function and promote hepatic tumor progression without causing significant lipid peroxidation [160].

In the context of renal studies, MPs/NPs, particularly PS-based particles, are emerging as environmental contaminants with increasing evidence of nephrotoxic potential. The kidney, a critical organ for metabolic waste elimination, appears especially vulnerable to MP/NP accumulation following diverse exposure routes. In murine models, autonomous inhalation of PS resulted in renal accumulation after pulmonary entry, with transcriptome analysis revealing persistent disruption of oxidative stress, inflammatory, and coagulation pathways, notably activating *NR4A1/CASP3* and *TF/F12* signaling [161]. Chronic oral exposure studies further indicate that environmentally relevant concentrations of PS and amino-functionalized PS (10 mg/L for six months) induce renal fibrosis through inflammation, inflammatory cell infiltration, and ferroptosis in renal tubular epithelial cells mediated by ferritinophagy and TGF-β1 secretion, effects reversible by the ferroptosis inhibitor Fer-1 [162]. Additionally, subchronic exposure to low-dose PS (0.1–1 mg/L for eight weeks) altered renal structure without affecting body weight or kidney coefficient, increasing glomerular tuft area and modifying transcriptomic profiles enriched in mitochondrial function, thermogenesis, and oxidative phosphorylation, with UQCR11 and MT-CO3 identified as key protein nodes [163]. Collectively, these studies demonstrate that MP/NP exposure—via inhalation or ingestion—can impair renal structure, function, and development, with mechanisms involving oxidative stress, ferroptosis, mitochondrial dysfunction, and inflammatory remodeling, underscoring their potential role as risk factors for kidney disease [164].

As indicated by the murine model, exposure to 1 μm PS has been demonstrated to induce significant hepatic damage via the activation of the p53 and p21 signaling pathways. According to recent findings, the transcription factor SALL2 has been identified as a probable oncogenic driver in the development of cancer induced by MPs/NPs [165]. Likewise, in mice, exposure to PS-NPs was found to accelerate the growth of epithelial ovarian cancer (EOC) and decrease cell viability in a dose-dependent manner, with effects linked to modifications in the tumor microenvironment and an increased mitotic index of malignant cells [166].

Respiratory effects have also been documented. Inhalation experiments in rodent models revealed that PS can become embedded in lung tissue, triggering inflammatory responses characterized by elevated secretion of proinflammatory cytokines, increased oxidative stress, and cellular damage, with consequent DNA disruption mediated by TNF-α, IL-6, and IL-1β signaling [167,168]. These cellular and genetic alterations potentially promote lung cancer progression following chronic exposure. Additional research is required to validate this association in human populations.

## 6. Prospective Considerations and Public Health Policy Frameworks

Longitudinal human studies are critically required to assess the impact of chronic MP/NP exposure on cancer progression through distinct molecular mechanisms, including chronic inflammation, oxidative stress, DNA damage, and epigenetic modifications. MPs/NPs warrant attention for their superior cellular penetration and possible effects on oncogenic pathways. It is imperative that research evaluates tissue-specific effects by considering routes of exposure (ingestion, inhalation, dermal contact), sources of material [169], environmental co-factors [170], and individual variables such as age, sex, genetic predisposition, and non-genotoxic tumor-promoting effects [171]. Moreover, investigations should comprehensively address the synergistic effects of MPs/NPs from environmental persistence and bioaccumulation. Additionally, there is a necessity to develop technologies for the detection and removal of MPs/NPs from human tissues.

### 6.1. New Research Directions

Advancing the understanding of MP/NP-associated carcinogenesis requires mechanistic studies on their bioaccumulation, tissue persistence, host–pathogen interaction, and interactions with cancer-related cellular pathways. Robust experimental models—including in vivo and in vitro systems mimicking human exposure—are critical to validate findings. Longitudinal studies and diverse experimental designs will improve risk assessment by clarifying contamination thresholds and health outcomes across MP types. Interdisciplinary integration of toxicology, epidemiology, and environmental science is essential to develop comprehensive exposure frameworks [172].

### 6.2. Policy Perspectives in Public Health

The rapid global plastic pollution crisis demands immediate public health action, with research underpinning policy development. Legislative measures to restrict plastic use and reduce MP contamination are gaining international traction, yet their efficacy requires evaluation through targeted studies. Rapid urbanization and economic growth exacerbate exposure risks, necessitating strategies to mitigate health impacts [173,174,175]. Research priorities include assessing MP/NP control in food systems, waste management innovations, and sustainable material alternatives to inform evidence-based regulations [55]. International collaboration is paramount to standardize risk assessment methodologies, harmonize testing protocols, and reduce plastic production, ultimately minimizing human exposure to these particles [176].

#### 6.2.1. Individual-Level Actions to Reduce MP/NP Exposure

Behavioral modification is a critical component in the effort to mitigate MP/NP exposure. Individuals can meaningfully diminish their MP/NP footprint through informed decision-making supported by accessible information and practical alternatives. Key strategies include limiting the use of single-use plastics, avoiding synthetic textiles, choosing natural personal care products, and prioritizing fresh or minimally packaged foods to reduce contact with plastic packaging—a major source of dietary MPs/NPs [115].

Practical lifestyle adjustments can further enhance these preventive efforts, including the adoption of alternative materials, such as glass or stainless-steel containers, for food storage purposes can mitigate the risks associated with ingestion. Installing household water filtration systems, including those using activated carbon or reverse osmosis, effectively removes MPs/NPs from drinking water [177]. Additionally, choosing clothing made from natural fibers over synthetic materials like polyester can minimize microfiber shedding during laundry, a major contributor to aquatic MP/NP pollution [178]. Simple interventions, such as using laundry filters or adjusting washing practices, have been shown to reduce domestic MP/NP release by up to 50% [179].

#### 6.2.2. Community-Level Actions: Role of Local Governments and Health Institutions

Local governments and health institutions play a pivotal role in mitigating MP/NP exposure and fostering sustainable behavioral change. Municipalities have the capacity to implement regulatory measures such as prohibiting single-use plastics and microbeads in personal care products. These measures can follow policies like the US Microbead-Free Waters Act of 2015. Additionally, municipalities can adopt deposit–return systems for plastic bottles or enact bans on plastic bags. These measures have been shown to be effective in reducing plastic waste [180,181,182]. Investing in efficient waste management and recycling infrastructures, as well as advanced wastewater treatment technologies like membrane bioreactors, is essential to prevent MPs/NPs from entering aquatic ecosystems [183]. Health institutions bear a significant responsibility in this regard. The integration of MP/NP risk assessment into environmental health surveillance systems is essential, and the establishment of monitoring programs to track MP/NP contamination in water, food, and air is strongly recommended. Findings should be shared with the public openly. It is imperative to educate healthcare providers in recognizing and addressing the potential health impacts of chronic MP/NP exposure, particularly among vulnerable populations such as workers in the textile or plastics industries [26]. Concurrently, educational strategies are imperative for cultivating early and long-term awareness. The integration of the MP/NP issue into educational curricula and public health initiatives has the potential to cultivate responsible consumer behaviors and environmental stewardship from an early age. Furthermore, educational institutions and places of employment have the capacity to implement “plastic-free” policies with the objective of diminishing environmental plastic shedding and exemplifying sustainable practices for the general community.

#### 6.2.3. Regional-Level Strategies: Cross-Border Cooperation and Harmonization

Regional coordination is critical for addressing MP/NP pollution, particularly in transboundary ecosystems such as rivers, lakes, and coastal zones. Countries that share natural resources must harmonize regulations, share environmental data, and implement coordinated monitoring programs to ensure the protection of shared environments. Regional economic and political alliances—such as the European Union (EU), Association of Southeast Asian Nations (ASEAN), and Pacific Alliance—can play a leading role in establishing binding agreements for MP/NP management, facilitating technology transfer, and funding infrastructure improvements in less-resourced member states. Initiatives like the EU Strategy for Plastics in a Circular Economy and the European Green Deal’s Circular Economy Action Plan serve as models for integrated action, including measures to reduce MPs/NPs from tire wear, packaging, and industrial sources [184]. These policies emphasize extended producer responsibility, product design standards, and reductions in primary MP/NP emissions from industries such as synthetic textiles. Harmonized regulations across borders, including standardized labeling for “MP/NP-free” products and penalties for non-compliance, can strengthen regional enforcement and compliance [185]. Moreover, regional research networks should coordinate studies on transboundary MP/NP flows and pollution hotspots, using shared data to guide policy. Regional health systems can collaborate to assess the health impacts of MP/NP exposure and develop targeted interventions. Public awareness campaigns implemented across multiple countries can further amplify the effectiveness of behavioral change strategies, ensuring a consistent message that transcends national borders.

#### 6.2.4. International Strategies: Governance, Research, and Cooperation

**Figure 4:**
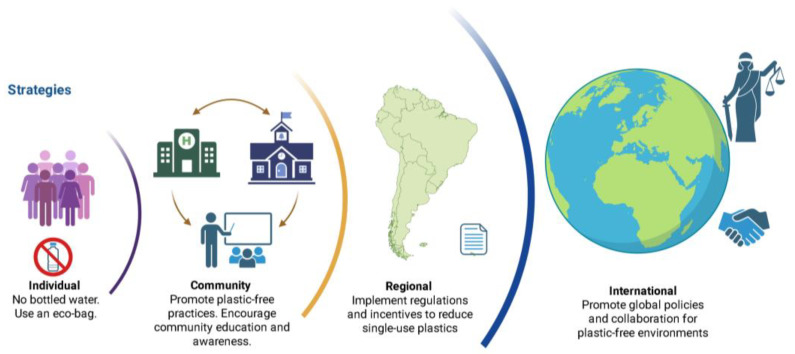
A multi-level strategy to reduce MP/NP pollution globally. The figure illustrates a progressive framework that begins at the personal level by promoting individual actions such as avoiding bottled water and using eco-bags. At the community level, the focus shifts to raising awareness and encouraging sustainable practices within local groups. Moving to the regional level, the emphasis is on implementing regulations and incentives to reduce single-use plastics. Finally, at the global level, the goal is to promote international policies and collaboration to mitigate MPs/NPs contamination worldwide as a new agreement. Created in BioRender. Ponce, R. (2025) https://BioRender.com/4szcpqa.

At the international level, coordinated frameworks and legally binding agreements are essential to address the transboundary nature of MP/NP pollution. The ongoing negotiation of the United Nations Treaty on Plastic Pollution, initiated in 2022, represents a landmark opportunity to regulate the entire plastic life cycle—including MPs/NPs—as part of a global environmental health agenda [186]. This treaty should explicitly classify MPs/NPs as hazardous pollutants, mandating global reductions in production and environmental release. International organizations such as the United Nations Environment Programme (UNEP) and the World Health Organization (WHO) must take leading roles in developing scientific guidelines, standardizing global monitoring systems, and advancing research on the health effects of chronic MP/NP exposure. The WHO should issue global exposure recommendations and support biomonitoring programs, particularly in low- and middle-income countries where environmental health infrastructure is limited [187]. Furthermore, the International Agency for Research on Cancer (IARC) should evaluate the carcinogenic potential of commonly used plastic polymers, potentially classifying them as Group 2B (possibly carcinogenic to humans), which could catalyze global policy action [188]. To ensure equity in implementation, a global financing mechanism—funded in part by the plastic manufacturing industry—should support the development of sustainable waste management systems, particularly in countries with limited resources. Promoting circular economy principles and funding research into biodegradable alternatives and safer plastic substitutes are also critical. Additionally, the creation of a “Global Microplastic Observatory” could serve as a centralized platform to monitor international progress, facilitate data sharing, and disseminate best practices across regions. All levels of action to minimize the impact of MPs/NPs are shown in Figure 4.

## 7. Conclusions

MPs/NPs represent a persistent environmental contaminant with growing evidence linking their exposure to cancer development and progression. This review consolidates current mechanistic insights demonstrating that MPs/NPs can induce oxidative stress, chronic inflammation, and genotoxic effects while also disrupting epithelial barriers, altering microbiota, and modulating tumor microenvironments across multiple organs. Their bioaccumulation in human tissues, coupled with the adsorption and delivery of hazardous chemicals, underscores their potential role in carcinogenesis. Preliminary findings from in vitro, in vivo, and epidemiological studies suggest the presence of organ-specific oncogenic pathways, particularly in the prostate, liver, lungs, breast, and gastrointestinal tract. However, critical gaps remain in understanding dose–response relationships, long-term health effects, and interactions with genetic susceptibility.

In summary, MPs/NPs represent a novel, multifaceted threat to human health. Although research on their carcinogenic potential continues, current evidence demands prompt scientific and regulatory review. The integration of MP exposure into cancer research, environmental health policy, and clinical oncology may pave the way for innovative preventive strategies and improve risk assessment models in the era of anthropogenic pollution. The establishment of a “Global MP/NP Observatory” has the potential to function as a centralized platform, providing a comprehensive and coordinated approach to the monitoring of international progress in the context of MP/NP pollution.

## Figures and Tables

**Table 1:** Physicochemical characteristics and cancer-associated effects of identified MPs/NPs.

Type of MPs/NPs	Reported Source or Context	Relevant Property	Cancer-Related Implication	Tissue/Cell lines	Particle Size	Observed Effects	References
PE	Cosmetics, packaging, textiles	Hydrophobic, low-density	Disruption of epithelial barriers and inflammation	Prostate, Cervical, Nervous, Skin	20–75 µm	Inflammation; cell proliferation and migration	[29,30,31,32,33,34,35,36]
PP	Containers, utensils	Thermally stable, resistant to degradation	Inflammation, metastasis	Breast, Cervical	0.5–4.5 µm, 20 µm	Cancer cell survival and growth; overexpression of metastatic genes	[37]
PS	Food packaging, foam materials	High surface adsorption potential	Oxidative stress, genotoxicity	Lung, Skin, Gastric, Liver, Colorectal	0.08 µm, 10 µm, 70 nm	ROS, mitochondrial damage, apoptosis	[23,25,38,39,40,41,42,43]
PET	Bottles, textiles	Aromatic structure, environmental persistence	DNA damage	Prostate, Colorectal	20–30 µm	DNA damage	[44,45,46]
PVC	Pipes, packaging, bags	Heavy metal release	Genotoxicity, endocrine disruption	Gastric, Liver, Breast	5 µm	Inhibition of repair mechanisms; genomic damage and metastasis	[47,48,49,50,51,52]
HDPE	Plastic materials	TGF-β signaling, NER disruption	Cell cycle and DNA repair disruption	Gastric	5 µm	Enhanced migration, ROS	[53]

**Table 2:** Analytical methods used for the identification and characterization of MPs/NPs in human tissues.

Analytical Techniques	Principle	Applicable Matrices	Strengths	Limitations	References
Micro-FTIR (Fourier Transform Infrared Spectroscopy)	Molecular vibration-based polymer identification	Placenta, lung, gastrointestinal tissue	Non-destructive, high specificity	Limited to particles >10 µm; interference from tissue autofluorescence	[61,137]
Micro-Raman spectroscopy	Inelastic light scattering for chemical fingerprinting	Lung, blood, feces	High spatial resolution; can detect particles <1 µm	Fluorescence interference; sample prep-sensitive	[112,138]
Pyrolysis gas chromatography–mass spectrometry and liquid chromatography–tandem mass spectrometry	Thermal decomposition followed by gas chromatography	Blood, tissues, urine	High sensitivity; quantification of polymers	Destructive; no info on particle shape/size	[139,140]
Scanning/Transmission Electron Microscopy (SEM/TEM)	Direct particle imaging	Human fibroblasts	Nanometric resolution	Requires metal coating or staining; poor chemical ID	[141]
Flow cytometry light scatter	Measures particle size via light scattering	Blood	Effective for nanoplastics (<1 µm)	Limited to liquid samples	[142]
Positron emission tomography (POET)	MPs/NPs tagged with stable isotopes or radiolabels	In vivo murine model	Enables biodistribution tracking	Currently limited to animal models	[143]
Confocal laser scanning microscopy (CLSM)	Visualization of fluorescent-labeled particles	Cell cultures, tissues	Allows localization in 3D; non-destructive	Only for labeled particles; autofluorescence interference	[32,144]

**Table 3:** Comparison of in vitro and in vivo studies evaluating carcinogenic effects of MPs/NPs.

Biological Mechanism	Experimental Support	Model Type	Level of Evidence	Findings
Oxidative stress induction	Demonstrated	In vitro (HeLa, HepG2), in vivo (murine) in vivo (zebrafish)	Strong	Well-documented generation of ROS and JNK and p38 MAPK activation [38]. Elevated catalase (CAT) and superoxide dismutase (SOD) [80].
Chronic inflammation	Demonstrated	In vitro (SCL-1 and A431)	Strong	NLRP3-mediated inflammation [32].
Disruption of epithelial barriers	Demonstrated	In vitro human kidney (HK-2) and testis (NTE) cells	Moderate–Strong	Claudin-2 tight junction protein loss [117].
Biofilm-mediated dysbiosis	Emerging	In vivo murine model	Moderate	Intestinal injury and gut microbiome dysfunction [145].
EMT induction	Demonstrated	In vitro (A549, colon cell lines)	Moderate	EMT mediated by NOX4-activated ROS [107].
Immune suppression (*TLR/NLR* pathways)	Suggested	Transcriptomic and protein expression data	Weak–Moderate	Some *TLR4/NF-κB* activation shown, but lacking full mechanistic tracing [146].
Genotoxic	Partially demonstrated	In vivo (hemocytes) in vitro (lymphocytes)	Weak–Moderate	Genotoxic properties only observed in vitro [147].
Genotoxin production via altered microbiota	Suggested	Mouse CRC models with microbiome modulation	Weak–Moderate	Presence of colibactin and DNA damage shown, but not fully attributed to MPs [148].
*STAT3* pathway activation	Speculative	In vitro (carp and mice)	Moderate	No direct studies linking MP exposure to *STAT3* in cancer contexts [149,150].
